# The Role of the Immune Response in *Chlamydia trachomatis* Infection of the Male Genital Tract: A Double-Edged Sword

**DOI:** 10.3389/fimmu.2014.00534

**Published:** 2014-10-27

**Authors:** Kate A. Redgrove, Eileen A. McLaughlin

**Affiliations:** ^1^Priority Research Centre in Reproductive Biology and Chemical Biology, University of Newcastle, Callaghan, NSW, Australia; ^2^School of Environmental and Life Science, University of Newcastle, Callaghan, NSW, Australia

**Keywords:** *Chlamydia*, infection, male fertility, inflammation, persistence

## Abstract

*Chlamydia trachomatis* (CT) is the most prevalent bacterial sexually transmitted infection in the world, with more than 100 million cases reported annually. While there have been extensive studies into the adverse effects that CT infection has on the female genital tract, and on the subsequent ability of these women to conceive, studies into the consequences on male fertility have been limited and controversial. This is in part due to the asymptomatic nature of the infection, where it is estimated that 50% of men with Chlamydia fail to show any symptoms. It is accepted, however, that acute and/or persistent CT infection is the causative agent for conditions such as urethritis, epididymitis, epididymo-orchitis, and potentially prostatitis. As with most infections, the immune system plays a fundamental role in the body’s attempts to eradicate the infection. The first and most important immune response to *Chlamydia* infection is a local one, whereby immune cells such as leukocytes are recruited to the site of infections, and subsequently secrete pro-inflammatory cytokines and chemokines such as interferon gamma. Immune cells also work to initiate and potentiate chronic inflammation through the production of reactive oxygen species (ROS), and the release of molecules with degradative properties including defensins, elastase, collagenase, cathespins, and lysozyme. This long-term inflammation can lead to cell proliferation (a possible precursor to cancer), tissue remodeling, and scarring, as well as being linked to the onset of autoimmune responses in genetically disposed individuals. This review will focus on the ability of the immune system to recognize and clear acute and persistent chlamydial infections in the male genital tract, and on the paradoxical damage that chronic inflammation resulting from the infection can cause on the reproductive health of the individual.

## Introduction

Sexually transmitted infections (STIs) are a major public health problem in most parts of the world, and are responsible for a number of acute illnesses, infertility, long-term disability, and premature death, in addition to contributing to an increase in the spread of HIV. It is estimated that $90–160 million is spent annually by the Australian health care system as a direct cost of *Chlamydia* treatment; this includes both initial treatment with antibiotics by a GP, and the more severe outcomes of untreated infection (1, 2). Perhaps the highest cost is associated with individuals who have troubles conceiving as result of infection and turn to assisted reproductive technologies (ART). In 2011 in Australia alone, a total of 61,158 ART treatment cycles were performed at a cost >$500 million (3). While there has been an increase in the promotion of *Chlamydia* prevention and screening programs, the largest barrier to reducing the rates of infection lies with the limited knowledge that people between the ages of 16 and 24 years possess, concerning the consequences, symptoms, prevalence, screening recommendations, testing procedures, and treatment of *Chlamydia* infection. It is, therefore, becoming essential that identification and treatment of *Chlamydia* infection is instigated before irreversible tissue damage occurs. Underpinning a successful public health program would be the development of a novel and effective chlamydial vaccine for young men. However, in order to undertake this, a thorough understanding of the intricate and often paradoxical immune response to *Chlamydia* infection in male reproductive tissues is needed. This review will highlight the known impacts that acute and chronic *Chlamydia* infection has on the male reproductive tract, as well as outlining some of the mechanisms that underlie the immune response in these unique tissues.

## Chlamydia

### Background and life cycle

*Chlamydiae* are obligate intracellular Gram-negative bacteria that are surrounded by a rigid cell wall. They are able to infect both human (*Chlamydia trachomatis* and *Chlamydia pneumonia*) and animals (*Chlamydia muridarum*, *Chlamydia suis*, *Chlamydia abortus*, *Chlamydia pecorum*, *Chlamydia psittaci*, and *Chlamydia caviae*) and depend entirely on the biosynthesis pathways of a host cell to multiply, as they are unable to synthesize essential nutrients (4, 5). For human *C. trachomatis*, there are 19 currently identified serotypes, determined based on their major outer membrane protein (MOMP) characteristics (6), with serotypes A, B, and C causing trachoma of the eye, serotypes D through to K infecting urogenital tissue (7), and serotype L being responsible for lymphogranuloma venereum (LGV), an infection of the lymphatics and lymph nodes (8, 9). Despite their common intracellular lifestyles, Chlamydia exhibits a range of hosts, as well as diversity in morphology, biological properties, and pathological consequences. The level of similarity for individual proteins encoded by *C. trachomatis* and *C. pneumonia* spans a wide spectrum (22–95% amino acid identity between orthologs from the two species) (10, 11).

**Figure 1:**
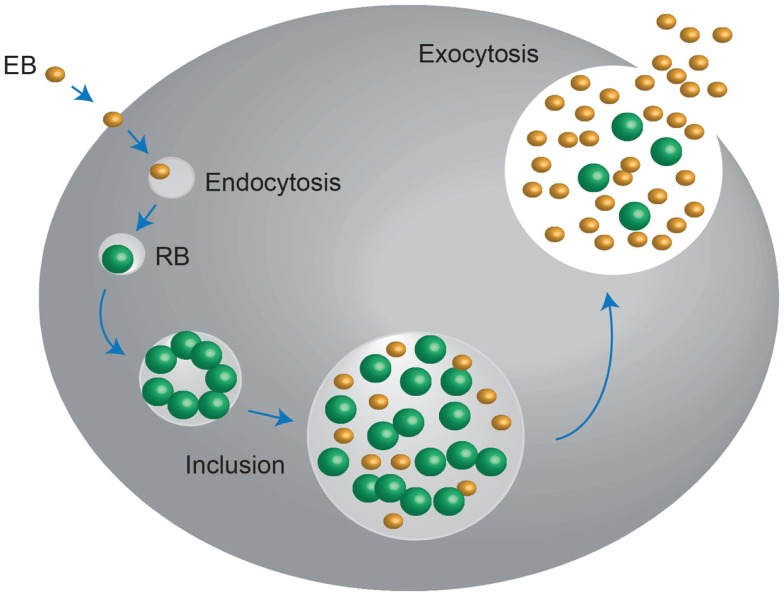
***Chlamydia* undergo a unique biphasic developmental cycle**. The infectious form of *Chlamydia*, the elementary body (EB) enters into the host cell via endocytosis. Upon entry, the EB convert into the metabolically active, non-infectious reticulate body (RB), which replicates within a vaculolar compartment, termed the inclusion. Once the developmental cycle is almost complete, the RBs revert back into EBs, stimulating host cell lysis and release of the infectious EBs into the extracellular space. These EBs then move onto to infect new host cells. Adapted from Roan and Starnbach (346).

*Chlamydia* exists in two developmental forms: the elementary body (EB), which is infectious, non-replicating, and extracellular. It also displays no metabolic activity; and the reticulate body (RB), which is non-infectious, replicating, and intracellular. Infection begins when the small (~0.2–0.3 μm) EB’s make contact with the epithelial cell surface. It has been proposed that number of receptor–ligand interactions take place at this point, after which the EB is endocytosed. The endocytic-vesicles are modified by the EB to prevent it from entering endocytic-lysosomal pathway and are then trafficked on cytoskeletal intermediate filaments to the endoplasmic reticulum/Golgi activity center. After arriving here, the transformation of the essentially non-metabolically active EB into the larger (~0.8 μm) metabolically active RB begins; the EB DNA is relaxed, signals for DNA, RNA, and protein synthesis are activated and RB cell division ensues. This intracellular Chlamydial micro-colony is now termed an inclusion, and after several hours of logarithmic RB growth, the inclusion expands. This, in conjunction with nutrient depletion and ATP scavenging from the infected host signals the transformation of the non-infectious RB back into the infectious EB, which are then exocytosed from the host cell to infect neighboring epithelial cells, in order to perpetuate the infection process (12–14) (Figure 1).

### Infection/interaction with host cells

*Chlamydia* are capable of invading the majority of cultured cells, which would suggest that the receptor(s) that facilitate the invasion are either ubiquitous, or that multiple receptors are used. However, the receptor–ligand interactions involved during chlamydial entry have proven to be elusive. This is in part due to the use of different species or strains of *Chlamydia* as well as different experimental procedures and parameters, making it difficult to draw comparisons between the multitudes of studies performed. It is thought that the binding may be a two-step process in some species, involving an initial, reversible, electrostatic interaction mediated by heparin-sulfate proteoglycans (HSPGs) (15–17), followed by high-affinity, irreversible binding to a secondary receptor (11). In addition to this, cleavage of the N-linked oligosaccharides, on the surface of *C. trachomatis* and *C. pneumonia*, inhibited attachment of the bacteria to a number of cell types, suggesting that glycan moieties of proteins expressed on the *Chlamydia* cell surface also participate in binding (18). Some of the proposed ligands that allow *Chlamydia* to attach to and infect host cells include the MOMP (16, 19), heat shock protein 70 (20), and glycosaminoglycans (GAGs) (15). Studies have also shown that lipopolysaccharide (LPS), one of the major components of the chlamydial cell surface, may play a role in the infectivity of *Chlamydia* into host cells, although the evidence suggests that the interaction may be complex and rely on more than one moiety (21). There has also recently been some interest generated by the entry of *C. trachomatis* into host cells via cholesterol-rich membrane domains, or lipid “rafts,” which appear to be serotype-dependent (22).

### Pathophysiology – males and females

Approximately, 75% of *C. trachomatis* infections in women and up to 50% of those in men are asymptomatic (23, 24). Clinical manifestations of *C. trachomatis* infections in women include acute urethral syndrome, urethritis, bartholinitis, cervicitis, upper genital tract infection (including endometritis, salpingo-oophoritis, and pelvic inflammatory disease), perihepatitis, and reactive arthritis (23). In women, untreated *C. trachomatis* infection can lead to severe reproductive complications. Pelvic inflammatory disease is a particularly common complication of chlamydial infection with consequences including infertility, ectopic pregnancy, chronic pelvic pain, and miscarriage (25–30). In fact, it is estimated that up to two-thirds of cases of tubal-factor infertility and one-third of cases of ectopic pregnancy can be attributed to *C. trachomatis* infection (31). In addition to this, chlamydial infection during pregnancy is associated with a number of adverse outcomes, including preterm labor, premature rupture of amniotic membranes, low birth weight, neonatal death, and post-partum endometritis (32, 33). Chlamydial infection can also be transmitted to the infant during delivery (34). An infant born to a mother with an active infection has an estimated 50–75% risk of acquiring infection. Indeed, approximately 30–50% of infants born to chlamydial-positive mothers will have chlamydial conjunctivitis, and at least half of these infants will also display nasopharyngeal infection. Further to this, it is common in about 30% of cases that infant with nasopharyngeal infection will also develop chlamydial pneumonia (23).

### Male genital tract infection and pathology

In men, *C. trachomatis* infection is known to be responsible for urethritis (35), epididymitis, epidiymo-orchitis (36–38), and it is becoming more widely accepted that it also acts as a causative agent for prostatitis (39–44), as well as causing an enlargement of seminal vesicles in the epididymis (37, 45–48), although the direct consequences of the *C. trachomatis* infection on the prostate and seminal vesicles remains unknown.

#### Urethritis

Urethritis is commonly defined as infection-induced inflammation of the urethra. The term is usually reserved for urethral inflammation caused by an STD and is normally characterized into gonococcal urethritis (GU), or non-gonococcal urethritis (NGU). Interestingly, many patients with urethritis, including approximately 25% of patients with NGU, are clinically asymptomatic (49). It is, therefore, unsurprising that *C. trachomatis* is a major cause of urethritis in men. Various studies have estimated that 30% of urethritis cases can be attributed to *C. trachomatis* infection (50). In addition to this, it is though that up to 42% of NGU cases may be caused by *C. trachomatis* (51). Importantly, *C. trachomatis* infection appears to be equally present in both symptomatic and asymptomatic urethral diseases (52, 53).

#### Epididymitis and epididymo-orchitis

Epididymitis and epididymo-orchitis are conditions exemplified by inflammation of the epididymis and testes, respectively, with or without infection. Indeed, according to the National Institute of Health, in the United States, where the population contains approximately 150 million males, 600,000 cases of epididymitis are recorded each year (38). The symptoms for epididymitis include pain, nodules, edema, urinary difficulties, fever, urethral discharge, and infertility (54). The condition can be classified based on the duration of symptoms as acute, sub-acute, or chronic. If the symptoms include pain and swelling, and desist within 6 weeks then the case is termed acute. If there is pain without swelling and the symptoms persist for longer than 3 months, then it is labeled as chronic (43). Epididymo-orchitis occurs when the inflammation from the epididymis spreads to the adjacent testicle (38). In men younger than 14 years and older than 35 years, epididymitis is generally caused by infection with a common urinary tract pathogen such as *Escherichia coli*. In those between 14 and 35 years, however, it is most commonly caused by sexually transmitted *Neisseria gonorrheae* or *C. trachomatis* (55, 56). Importantly, decreased sperm counts and decreased motility are often consequences of acute epididymo-orchitis (57), and this pathology is also associated with high rates of infertility (58).

#### Prostatitis

Prostatitis is a state of inflammation of the prostate, which can be described both with and without infection. Prostatitis syndromes can be divided into four different classifications: (I) acute, (II) chronic, (III) non-bacterial prostatitis, chronic pelvic pain syndrome (CPPS), and (IV) asymptomatic inflammatory prostatitis (59). Although acute and chronic prostatitis have a clear etiology and patients respond well to anti-microbial treatment, these types of prostatitis only encompass 10% of the cases seen in clinical practice (60). CPPS is the most common prostatitis syndrome, constituting 90–95% of cases. Patients with CPPS have no evidence of urinary tract infection, making it a common disease of unclear etiology (60). There have been a number of studies highlighting the prevalence of *C. trachomatis* infection in patients with prostatitis (9, 39, 40, 42, 44, 46, 61–66). The rates of prevalence, however, are variable between studies, and have been related back to differences in the types of samples analyzed, for example, urethral swab, first void urine, semen, or expressed prostate secretion. In addition to this, concerns have been raised about the reliability of the samples used in these studies. In particular, it has been postulated that bacterial isolation from urethral swabs, expressed prostatic secretions (EPS), and/or urine following prostatic massage, have the potential to be contaminated as a result of transiting through the urethra, thus limiting the interpretations that can be made from such tests (64, 67). However, a number of studies indicate that semen/EPS specimens are often positive for *C. trachomatis* in patients with negative urethral swabs (40, 63, 66, 68). Moreover, pure prostatic biopsies from CPPS have identified the presence of *C. trachomatis* in the absence of urethral infection (39, 42). It is thought that *C. trachomatis* infection of the prostate gland may cause inflammation and thus impair the normal functionality of the gland and impact on male fertility (69). As stated above, the literature concerning this issue is controversial, with some reports arguing in favor of a positive relationship between chronic prostatitis induced by *C. trachomatis* and altered semen quality (70–73), whereas other reports support the concept that no alterations in semen quality and male fertility are observed (46, 68, 74–79).

#### Seminal vesiculitis

Seminal vesiculitis is inflammation of the seminal vesicles, and is most often a secondary outcome of prostatitis, although it is also know to occur independently. It is still uncertain in human beings whether *C. trachomatis* can infect seminal vesicles and lead to inflammation and a specific pathology. This is mainly due to the lack of clinical symptoms and/or significant consequences that the infection produces (80). However, studies performed by Furuya and colleagues reported the presence of inflammation in the seminal vesicles of patients with acute epididymitis, and that *C. trachomatis* was the pathogen most frequently detected in the seminal vesicle fluid (81). In addition, vesiculitis-associated symptoms disappeared simultaneously with improvement in symptoms of epididymitis after anti-microbial treatment (81). These finding strongly suggest that seminal vesicles are involved in the urogenital inflammation process. Furthermore, some researchers have proposed that chlamydial epididymitis may originate from seminal vesiculitis (37). This is supported by a reported case of seminal vesiculitis appearing prior to acute epididymitis in a patient whose female partner had been diagnosed with chlamydial cervicitis (82). It has also been shown that patients with urethritis are more likely to have accompanying seminal vesiculitis (83).

### Chlamydia and male infertility

While it is clear that the inflammation caused during chlamydial infection has a direct effect on the male reproductive tract itself, it remains largely controversial as to whether infection with *Chlamydia* has a dramatic effect on sperm quality and subsequent male infertility. Studies using electron microscopy have demonstrated an interaction between *Chlamydia* and sperm in biopsies taken from both testis and epididymis, in addition to semen samples (84, 85). In addition to this, a study using transmission electron microscopy showed that specific *C. trachomatis* serotypes (86). More conclusive evidence for negative effects of *Chlamydia* infection on male fertility is offered in a number studies that demonstrate that the EBs of *C. trachomatis* can lead to apoptosis of human sperm *in vitro*, via the activation of specific caspases (87–89). It is well known that this effect can be elicited by *Chlamydia* LPS (90, 91). Based on this evidence, it has been proposed that chlamydial LPS interacts with CD14 on the surface of the sperm leading to an increased production of reactive oxygen species (ROS), subsequently activating apoptotic caspases (92). Importantly, excessive generation of ROS has been correlated with an increase in sperm defects both *in vitro* (93) and in infertile men (94). In support of the detrimental effects of infection on male fertility, a number of studies have demonstrated that *C. trachomatis* infection correlates with reduced sperm motility (95–98), increased proportion of sperm abnormalities (99), a significant reduction in semen density, sperm morphology and viability (100), and an increase likelihood of leukocytospermia (98, 101). Co-infection with *Chlamydia* and *Mycoplasma* results in over threefold more sperm cells presenting with fragmented DNA than uninfected controls (98). In contrast to this, however, there have been multiple studies that suggest that positive markers for *C. trachomatis* infection are not associated with altered sperm parameters and quality (46, 68, 74–79, 102). A role for *C. trachomatis* in male factor infertility is also yet to be proven (24, 31). A number of studies have attempted to prove an association between infection and infertility. For example, Mosli and colleagues examined chlamydial incidence via direct immunofluorescence and culture of urethral swabs in age-matched partners of infertile couples (MPIC) and fertile controls, and demonstrated rates of *Chlamydia* infection to be 25 and 4%, respectively (103). However, it is difficult to compare these results with other studies performed due to the variance among the studies including the differences in patient demographics, the methods of *Chlamydia* diagnosis used and the samples that were examined (43).

It has been estimated that 5–10% of male factor infertility can be attributed to inflammatory or autoimmune responses, including orchitis, epididymitis, and epididymo-orchitis, as discussed above. However, an additional etiology caused by these immune responses is the production of anti-sperm antibody (ASA) formation. Indeed, ASA can be found in the seminal plasma or attached to the sperm surface in 5–12% of infertile men (104).These ASAs function to impair sperm fertilizing ability including affecting motility (105, 106), the ability to undergo a successful acrosome reaction (107), penetration of the cervical mucosa (108), binding to the zona pellucida (109), as well as sperm-oocyte fusion (110). These antibodies directed against sperm antigens can be found in the seminal fluid and seminal plasma in men, as well as in the follicular fluid in women. They can also be detected in the blood serum of both men and women (111). However, only those antibodies that are bound to the sperm are considered to have an effect on fertility (108, 112).

While all of the studies performed into the direct effects that *Chlamydia* infection may have on the fertility of untreated males, it is also important to consider the role that the immune response toward the bacterial infection may play toward creating the observed pathophysiology. While the biochemical environment, and immune response, of reproductive organs such as the prostate and the epididymis closely relate to those of mucosal tissues, the micro-environment that the developing sperm exist in within the testes is immunologically privileged. Indeed, developing sperm cells are produced long after the immune library has been established, and harbor specific antigens that cannot be found in any other organ, tissue, or cell within the body. As a consequence of this, these developing cells and the resultant spermatozoa are deemed to be “foreign” by the male immune system. However, the immune privileged environment, they experience within the testis and the epididymis aid in preventing an immune response (113, 114). In addition to this, foreign cells are able to exist within the testes without the induction of a large-scale immune response (115–117).

### Testis as an immune privileged site

#### Structure of the testis

**Figure 2:**
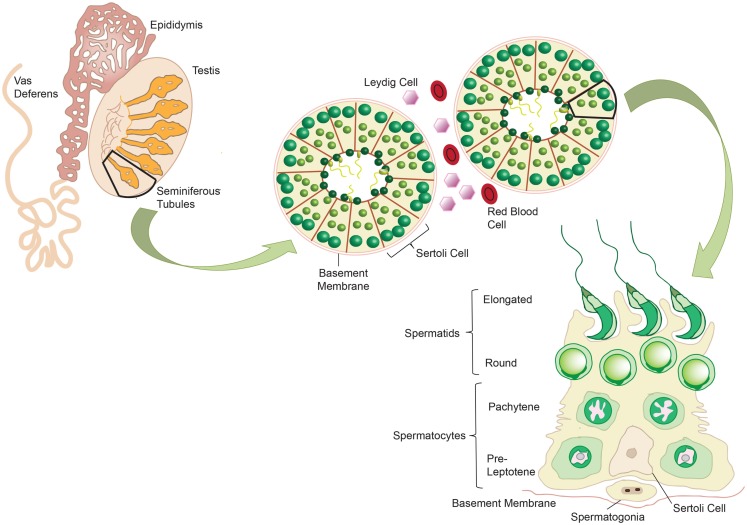
**Mammalian spermatogenesis occurs within the seminiferous tubules of the testis, with development of the mature sperm cells occurring in a radial fashion from the basement membrane into the lumen**. Spermatogonia reside on the basement membrane and undergo mitotic division to produce pre-leptotene spermatocytes. These primary spermatocytes undergo meiosis to give two pachytene spermatocytes, which in turn undergo meiosis to give round spermatids. These cells elongate and develop into mature spermatozoa. Sertoli cells support the germ cells as they develop, providing essential nutrients. Adapted from ‘Spermatogenesis Online’. Reproduction Data Systems 2011–2012. mcg.ustc.edu.cb/sdap1/spermgenes

**Figure 3:**
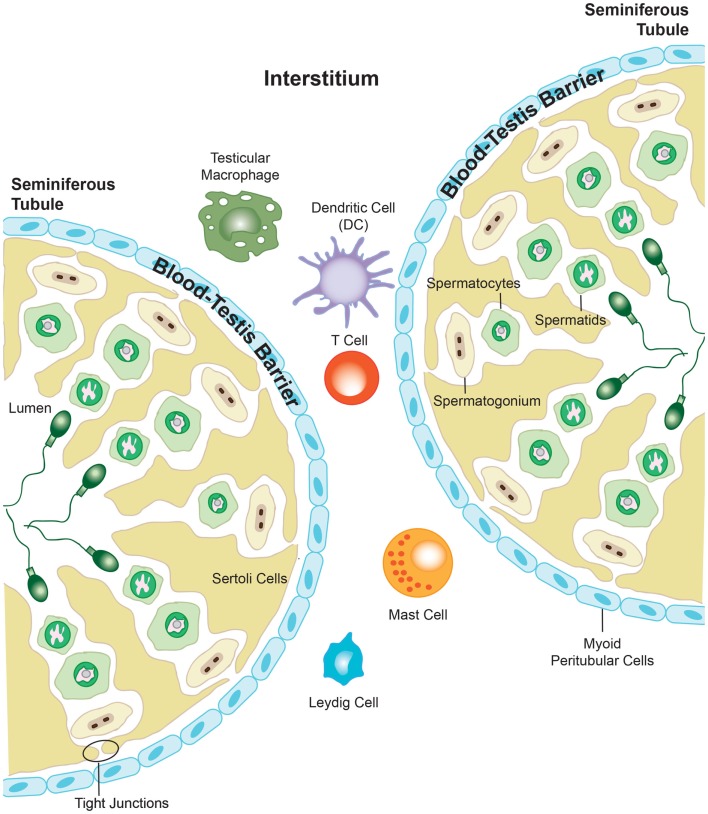
**The presence of the blood–testis barrier creates a region of immune privilege within the testis**. At the onset of puberty, developing sperm cells express novel antigens that the immune system would normally identify as “foreign.” However, segregation of antigens in the seminiferous tubules from the immune cells that are able to enter into the interstitial space of the testis prevents the body from eliciting an immune response against these vulnerable cells. Adapted from ‘Blood-Testis Barrier’. Immunopaedia.org 2010. www.immunopaedia.org.za/index.php?id+668

The testes fulfill two major functions for male reproduction: the first is to produce morphological mature and functional spermatozoa, and the second is the production and controlled release of sex steroids (primarily androgens). The testis is compartmentalized both histologically and functionally into two distinct regions to accommodate the two separate functions. Spermatogenesis takes place in the seminiferous tubules, while androgens are synthesized in the Leydig cells in the interstitial compartment that is dispersed between the tubules. The seminiferous tubules are tightly coiled, and originate and terminate at the rete testis. Each tubule is surrounded by myoid pertitubulur tissue that, together with the Sertoli cells, provides structural support, and secretes the components of the basal membrane enclosing the seminiferous epithelium. The columnar Sertoli cells extend from the basal lamina toward the lumen of the tubules, and are responsible for the physical support of the developing germ cells, as well as providing essential nutrients and growth factors (Figure 2). The most prominent components of the interstitial space are the Leydig cells that work to produce steroid hormones. The interstitium also contains blood vessels and immune cells, such as macrophages, dendritic cells (DC), lymphocytes and increasingly with age, mast cells (118) (Figure 3). Also studies performed by Holstein and Davidoff have described the presence of large, flat fibroblastoid cells, which compartmentalize the microvessels, the Leydig cells and part of the seminiferous tubules (119). These cells, termed “co-cells” (abbreviated from connective tissue cells/compartmentalizing cells/covering cells), appear to produce extracellular matrix components such as decorin, vimentin, and fibroblast surface protein (120), and are typically found only in human testis. The variability in the extracellular matrix components observed is important for cell–cell interactions within the testis (121). Some of these proteins are able to bind to various types of growth factors, creating a reservoir from which the bioavailability of these growth factors can be modulated (122).

#### Blood–testis barrier

The blood–testis barrier is formed by tight junctions between neighboring Sertoli cells localized in the seminiferous epithelium, and function to restrict the movement of molecules through the intracellular spaces (Figure 3). The barrier divides the seminiferous tubule into two distinct compartments: the basal compartment, which contains spermatogonial stem cells and early stage spermatocytes, and the adluminal compartment containing meiotic pachytene and secondary spermatocytes, in addition to haploid spermatids. The importance of the blood–testis barrier in creating immune privilege was first demonstrated using Sertoli cell-depleted, androgen-receptor knockout mice. The integrity of the tight junctions that form the blood–testis barrier was compromised in these mice, and as such, immune molecules were able to pass into the adluminal compartment and mount an attack on the spermatocytes and spermatids held within. This lead to an arrest in spermatogenesis, and it was proposed to have downstream negative effects on fertility (123). However, it is well known that the blood–testis barrier alone cannot be wholly responsible for the immune privileged status that exists within the testis, as germ cell auto-antigens have been shown to be expressed in the basal compartment and in spermatogonia and early spermatocytes, which are not protected by the blood–testis barrier (124, 125). As described previously, the blood–testis barrier is incomplete in the rete testis, a location where large numbers of morphologically mature spermatozoa expressing newly adapted surface molecules, move toward the epididymis (126, 127). Furthermore, Head and Billingham demonstrated that allografts placed in the interstitial space outside the blood–testis barrier, could survive for an extended period without experiencing immune rejection (117). They concluded that mechanisms additional to the physical barrier must be in place to maintain immune privilege in the testis. The blood–testis barrier terminates at the rete testis, and subsequently, spermatozoa are no longer protected from immune attack, which is confirmed by the observation that various forms of autoimmune orchitis manifest first in the rete testis (126–129). Spermatozoa move from the rete testis into the epididymis, which consists of a long, convoluted duct, and gain the capacity for fertilization. The epididymis also contains a blood–epididymis barrier, although this barrier is more functionally related to those found in other epithelia, with fewer exclusively apical tight junctions (130–132). In contrast to the seminiferous epithelium, T-lymphocytes and macrophages are frequently found within the epididymis epithelium and in the lumen of the epididymal duct (133–135), suggesting that the epididymis operates within a different immune environment to that of the testis.

## Immune Response

### Innate immunity

The first line of defense from chlamydial genital infection is the mucosal barrier of the genital tract. However, upon entering the mucosal lining and establishing a productive infection, it is the innate immune system that provides the next stage of defense against the bacteria. Although epithelial cells, which are the initial targets for *Chlamydia* infection, are not considered to be a part of the classical innate immune system, they are capable of initiating and sustaining innate immune responses (136). It is well known that *C. trachomatis* infection in both human beings and murine epithelial cells can induce the production of pro-inflammatory cytokines such as interleukin 1 (IL-1), Il-6, and tumor necrosis factor alpha (TNFα) (137, 138). In addition, secretion of chemokines such as Il-8 by infected cells can recruit classical innate immunity cells such as natural killer (NK) cells and DCs, which are abundant in the genital mucosa (139). However, as discussed above, the testis as an immune privileged site differs in the way the immune response develops against foreign pathogens, and thus warrants further exploration.

#### Neutrophils and natural killer cells

Neutrophils are the most predominant form of white blood cell, and have the dual functions of immune surveillance and *in situ* elimination of microorganisms (140). NK cells are classified as being cytotoxic lymphocytes that play a similar role to that of neutrophils (141). Importantly, NK cells and neutrophils are the first immune cells that are recruited to the site of chlamydial infection. It is thought that neutrophils work to reduce direct chlamydial infection and limit spreading, with human neutrophils being able to effectively inactivate *C. trachomatis in vitro* (142, 143). Additionally, mice that were neutrophil depleted had up to a 10-times greater burden of *C. muridarum* in the female genital tract than neutrophil-competent controls. However, both sets of mice were able to effectively eliminate the infection within the same period of time (144), which suggests that neutrophils are not critical for the resolution of the infection. As neutrophils are usually the first immune cells recruited to the site of infection, and are generally short-lived (145, 146), it is likely that the primary role for neutrophils is to reduce chlamydial infection and to limit it from spreading. In the context of the testes, the short-life span of the neutrophils is particularly important, as these cells are a major source of tissue-damaging cytokines, such as matrix metalloproteinase 9 (MMP9), during acute infection (147), and a prolonged life span for these cells may contribute to fibrosis and infertility. In support of this, recent evidence has emerged that indicates that *C. trachomatis* may delay neutrophil apoptosis, prolonging their life span (148). It remains unclear whether neutrophils carry out the same function in the clearance of *Chlamydia* from the immune privileged space of the testis, although their presence has been documented in the rat testis (149), and they have been shown to accumulate in the interstitium of mouse testis 9–12 h following exposure to *E. coli* (150). NK cells are more traditionally known for the role they play in viral infections and cancers. However, they have also been shown to be important in the early immune response and subsequent elimination of bacterial infections (151, 152), are their activity becomes enhanced in the presence of cytokines such as IL-12 and interferon γ (IFNγ) (Figure 3). While it has been shown the NK cells exist in the testis, their specific function remains unknown, although it is assumed that they undertake a traditional role in virus and tumor surveillance (153).

#### Dendritic cells

**Figure 4:**
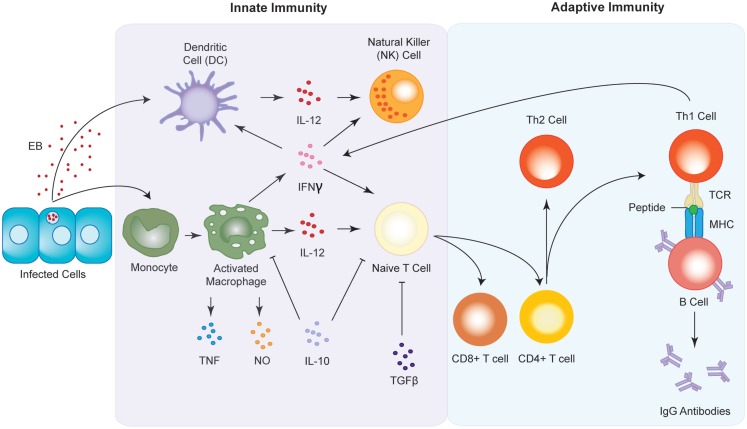
**Innate and adaptive immune responses to *Chlamydia* infection**. Upon infection, antigen presenting cells (APC) such as macrophages and dendritic cells are sequestered to the site of infection where they begin to release pro-inflammatory cytokines such as IFNγ and IL-12. The chemokines in turn activate natural killer (NK) cells and induce the maturation of T cells into either CD8^+^ or CD4^+^ T cells. CD4^+^ T cells go on to form either T-helper 1 (Th1) or T-helper 2 type (Th2) T cells. Th1 cell interact with B cells via the T cell receptor (TCR) and the major histocompatibility complex (MHC) to produce antibodies against the chlamydial infection.

Dendritic cells are recognized as being the archetypal antigen presenting cells (APCs). DCs migrate as immature or precursor cells from the bone marrow into peripheral tissues, whereupon they receive activation signals associated with inflammation or pathogen invasion. Once activated, they migrate to the local lymph nodes, where they mature and phagocytose the antigen. Once internalized, the DCs degrade the components of the antigen and present their peptides to T-cells via major histocompatibility complex (MHC) receptors, which activate the T-cells to initiate a cell-mediated and/or humoral immune response (Figure 4). DCs also have the ability to tolerize T cells to antigens, thereby minimizing aggressive autoimmune responses (154). Immature DCs have increased capacity to internalize antigens, but low T cell stimulatory activity, while mature DCs reciprocally down-regulate their ability to endocytose antigens but have high functioning T lymphocyte stimulatory capabilities (155). In addition to this, mature DCs express surface T cell stimulatory molecules such as CD40, CD80, and CD86, as well as MHC class II molecules. They also produce bioactive IL-12 and TNFα, and display altered migratory behavior (156), and are ultimately potent stimulators of immune responses. In contrast to this, those DCs in a resting state have been implicated in the creation of self-tolerance (157), with the presentation of “self” antigens on the surface of DCs thought to play a significant role in the initiation of autoimmunity, and its progression toward autoimmune disease (158, 159). Further to this, as DCs are APCs, they are not capable of targeting specific antigens, but instead present a wide range of antigens on their cell surface, including what have been labeled as danger signals. The working hypothesis, or “danger model” suggests that stressed or damaged cells and tissues express and release heat shock proteins (Hsps) during injury caused by trauma, inflammation, pathogens, or toxins. Recently, it has been shown that these Hsps act as testicular autoantigens, and may provide a mechanism for how DCs in the testis participate in the activation of lymphocytes and the subsequent damage of testicular tissue (160).

Both MHC class I and II molecules are expressed in the interstitial tissue of testis, including on macrophages and Leydig cells. In addition, DCs also express MHC class II molecules in the testis (161). Interestingly, developing germ cells fail to express MHC antigens on their surface, perhaps giving an indication of how these cells avoid detection by CD4^+^ and CD8 ^+^ T cells (162–168). However, despite the importance they play in regulating the immune response in other tissues, very few studies have investigated DCs in the testis. Cells that express DC markers or possess DC-like morphology have been observed in the testes of mice (169, 170), rats (117), and human beings (165, 171). Although these markers are also expressed on macrophages, the positive identification of these cells as DCs remains difficult. Nevertheless, co-stimulatory molecules such as CD80 and CD86 are expressed in the testis of 14- to 22-week-old non-obese diabetic mice (172), as well as in the rat testis (161), which suggests that DC-dependent activation of T-lymphocytes via binding to their specific antigens is at least possible in rodent testis.

#### Sertoli cells

Sertoli cells are somatic cells that, as described above, provide essential support for developing sperm cells in the testis and have a critical role in the establishment and maintenance of immune privilege. A number of studies have demonstrated that Sertoli cells possess the ability to act as immunosuppressants (173), and are capable of being transplanted into a variety of tissues (174–176) and inducing immune tolerance (173). In addition to this, these cells are known to possess characteristics more traditionally displayed by immune cells, and thus are thought to play an essential role in the immune response within the testis. They are capable of phagocytosing apoptotic spermatogenic germ cells (177), producing anti-microbial proteins (178–180), and perhaps most importantly express pattern-recognition receptors (PRRs) such as Toll-like receptors (TLRs) (181–184). TLRs operate by recognizing pathogen-associated molecular patterns (PAMPs) and structural subunits, including those for microbial cell walls (e.g., peptidoglycan), cell membranes (e.g., LPS), and virulence proteins (e.g., flagellin) (185), that are absent in the host. Upon recognizing these PAMPs, the TLRs become activated and induce production of pro-inflammatory cytokines, as well as cell adhesion molecules that recruit macrophages, neutrophils, and NK cells, and invoke maturation of DCs. They also secrete specific anti-microbial products such as interferons and defensins (186). To date, 10 TLRs have been identified in human beings, and 13 in mice (187), and the roles that each TLR plays depend on which PAMPs it recognizes. TLR2, in conjunction with TLR1 and TLR6 recognize various bacterial components (peptidoglycans, lipopeptide, and lipoprotein of Gram-negative bacteria, and lipopeptide of mycoplasma) (188, 189). TLR3 recognizes the dsRNA that is produced by replicating viruses (190). TLR4 in association with the co-receptor CD14 and the extracellular molecule MD-2 recognizes LPS (191, 192). TLR5 recognizes a highly conserved structure that is specific to bacterial flagellin (193). The presence of TLR2 and TLR4 on Sertoli cells has been demonstrated in both prepubertal and adult testis, with TLR5 and TLR6 also detected at lower levels. Importantly, it has been shown that the presence of IFNγ or TLR agonists induces a significant increase in TLR2, TLR4, and TLR6 mRNA, and TLR2 protein becomes up-regulated (181). Further to this, stimulation with pro-inflammatory cytokines resulted in Sertoli cells being able to bind lymphocytes, as well as secrete IL-6, indicating the potential to promote inflammation in the testis (181).

#### Interferon γ

Interferon γ plays a functional role in both the innate and adaptive immune response (Figure 4). It has the dual responsibility of inhibiting the growth of *Chlamydia* (194), as well as being one of the main cytokines important for type 1 T-helper cell (Th1) immune response (195). IFNγ is a cytokine produced by NK cells, as well as CD4^+^ and CD8^+^ T-cells, in response to signals such as Interleukin 2 (IL-2), basic fibroblast growth factor (bFGF) and epidermal growth factor (EGF). The importance of IFNγ *in vivo* has been shown through enhanced levels of bacterial infection in IFNγ^−∖−^ or IFNγ-receptor^−∖−^ mice, compared to controls (196–200). However, mice are able to resolve chlamydial infection in the absence of IFNγ, suggesting that while beneficial in the process of clearing chlamydial infection, it is not essential, and that there are T-and B-lymphocyte dependent mechanisms also playing roles. Indeed, studies using double-knockout mice null for both IFNγ (IFNγ^−∖−^) and T-and B-lymphocytes (RAG^−∖−^), demonstrated that when infected with *C. pneumonia*, the null mice displayed a higher rate of mortality, when compared with IFNγ^−∖−^ and RAG^−∖−^ mice single KO mice (201). IFNγ has been shown both in cell culture and *in vivo* models to provide protection against infection by *C. muridarum* and *C. psittaci* (202–204). The mechanism of action of this inhibitory effect is not the direct result of the IFNγ acting on the *Chlamydia* but instead due to alterations made to the host cell physiology and environment that impact on the ability of the *Chlamydia* to grow and replicate.

IFNγ affects human host cells *in vitro* by inducing the expression of indoleamine 2, 3-dioxygenase (IDO), an enzyme that catalyzes the initial step in the degradation of the amino acid tryptophan to N-formalkynurenine and kynurenine (205). IDO has been shown to mediate potent immunosuppression in classical immune responses, as well as fetal tolerance, tumor resistance, regulation of autoimmune responses, and maintenance of immune privilege in the epididymis (206–211). Depletion of exogenous intracellular tryptophan by IDO has been shown to starve the *Chlamydia* of an amino acid essential for its ability to differentiate into infections EBs (194, 203, 212). However, there are chlamydial species, which have successfully adapted to tryptophan starvation by transforming into a unique non-replicating but viable form (213). Following removal of IFNγ from the host cell, and subsequent resumption of tryptophan synthesis, these unique forms quickly differentiate back into infectious EBs and continue the infection. This cycle is known as a “persistent” infection, and is discussed in more detail in below.

Furthermore, it is well established in murine cells that IFNγ also activates inducible nitric oxide synthase (iNOS), an enzyme that catalyzes the production of anti-microbial reactive nitrogen intermediates, including nitric oxide (NO), from l-arginine (214). There have been a number of studies showing a correlation between the inhibition of intracellular chlamydial growth and the induction of NO secretion (214–219), suggesting that control of chlamydial infection in mice may involve the activation of the cytokine-iNOS system. In support of this, a recent study has linked IL-17A and IFNγ, suggesting that they work in a synergistic fashion to up-regulate iNOS, and subsequently NO production, in order to inhibit chlamydial growth.

Iron has also been shown to be important for *Chlamydia* survival (220–222). It is thought that the ability of IFNγ to down-regulate transferrin receptor (223–226), which regulates the import of iron into the cell, may limit the availability of the amount of intracellular iron available to the bacterium, thus limiting its growth. This has been shown to be true for other bacterial species such as *Legionella pneumophila* (225) and *Salmonella typhimurium* (227). In addition to this, IFNγ is able of enhancing the phagocytic capabilities of macrophages, and may promote the engulfment and subsequent elimination of *Chlamydia* (228, 229). It has been known for some time that IFNγ is produced by the Sertoli cells, peritubular cells and, at low levels, early spermatids within the testes (230). It has also been shown to influence testicular germ-cell tumors (TGCTs), paradoxically having either anti- or pro-apoptotic activity depending on the tumor type. Within these tumors, IFNγ bind to its receptor (IFNγ R) and activates the JAK/STAT (Janus kinase/signal transducer and activator of transcription) pathway. JAKs that have been activated by IFNγ phosphorylate STAT1 proteins, which are translocated to the nucleus, resulting in the transcriptional activation of specific target genes. Several of these genes have been identified, but the gene of note that is shown to be activated by IFNγ is that of interferon regulatory factor 1 (IRF-1) (231). IRF-1 mediates a diverse range of functions, including tumor suppression, myeloid differentiation, macrophage activation, antigen presentation, and T- and B-cell differentiation (232). In addition to this, studies by Kanzaki and colleagues has demonstrated the presence of IFNγR in rat Sertoli cells and that it also regulates the expression of the IRF-1 gene in the testis, potentially as a means of regulating the apoptosis if neoplastic germ cells (233). Importantly, interstitial macrophages also express high levels of IFNγ and TNFα during testicular inflammation, and that abnormal elevation of these cytokines has been associated with reduced fertility in inflamed testes (234). A recent study by Gao and colleagues further indicates that IFNγ works synergistically with TNFα, having pronounced disruptive effects on the blood–testis barrier and Sertoli-germ cell adhesion in the seminiferous epithelium, resulting in reduced fertility (235).

In the epididymis, however, it appears that IFNγ plays a minor role in the establishment and maintenance of the immune response. Although IDO is highly abundant in the epididymis, its expression is constitutive, and is IFNγ-independent. It is, therefore, thought that the epididymis is constantly in an inflammatory state, as IDO is considered to be a component of the early immune response to inflammation and infection (236).

#### Macrophages

Macrophages are phagocytic immune cells produced through the differentiation of monocytes and are central for the induction of the innate immune response. They are also APC, equipped with PRRs that aid in the recognition of various moieties from pathogens termed PAMPs, in addition to danger-associated molecular patterns (DAMPS) (237). During *Chlamydia* infection, macrophages migrate to the infected site (238), phagocytose the bacteria (239), and depending on the specific receptor-PAMP/DAMP match, induce various downstream effectors and pathways to produce pro-inflammatory cytokines (240, 241). Macrophages are activated by IFNγ, which is produced by CD4^+^ and CD8^+^ cells, and works to convert resting macrophages into potent cells with increased antigen presenting capacity, increased synthesis of pro-inflammatory cytokines and toxic mediators, and augmented complement-mediated phagocytosis (Figure 4). Further to this, the destruction of *Chlamydia* inside macrophages has been associated with autophagy, a process by which cells degrade cytoplasmic proteins and organelles (242–244). Studies have also demonstrated that macrophage autophagy can enhance antigen presentation to T cells (245). It is now well known that the macrophages make up the majority of the immune cells found within the testis (118, 246). Under normal conditions, macrophages, and indeed all other leukocytes, are found exclusively in the interstitial space. It is only under pathological conditions that macrophages are able to enter the germ cell compartment where they are capable of phagocytosing degenerating germ cells. In addition to possessing features common to all macrophages, testicular macrophages also appear to play an important role in male reproductive function. Indeed, it has been shown that macrophages within the testis exist in direct contact with Leydig cells, forming specialized contact sites known as digitations (247, 248). Furthermore, they are involved in Leydig cell development and the regulation of steroidogenesis in adults (249–253). The high levels of macrophages present within the testis are almost exclusively regulated by a Leydig-cell-mediated mechanism, with testosterone and macrophage-migration inhibitory factor (MIF) playing only minor roles, if any at all (254). The evidence for the close relationship between testicular macrophages and Leydig cells is represented by the loss of approximately 50% of these macrophages in Leydig-cell-depleted testis (255). These macrophages are also capable of influencing Sertoli cell function, and subsequent spermatogenesis through the release of soluble mediators (256).

It is well-established testicular macrophages are responsible for establishing and maintaining the immune response within the testis. Macrophages from normal tissues are APCs, with the ability to direct the outcome of T cell responses, and the expression of major histocompatibility (MHC) class II expression on the testicular macrophages suggests a similar role for these cells (118). However, the antigen presenting capabilities of testicular macrophages have yet to be fully elucidated, although it is known that they suppress both T cell responses *in vitro* (257) and *in vivo* (258). Interestingly, a number of studies have shown the testicular macrophages are significantly poorer at producing pro-inflammatory cytokines, as well as IL-1β and TNFα, although the production of prostaglandins and other cytokines are not inhibited (257, 259–261). However, despite this limitation in testicular macrophage function, the testis is still able to support an inflammatory response. Evidence suggests that testicular somatic cells such as Leydig cells, Sertoli cells, and peritubular cells, are capable of producing various pro-inflammatory mediators such as MIF, iNOS, and various isoforms of IL-1 (113, 262–268). In the rat testis, at least two subsets of macrophages have been identified, one which is recognized by the monoclonal antibody ED1, and the other which is a surface antigen recognized by the monoclonal antibody ED2 [reviewed by Hutson (269), Hedger (246), Hedger and Meinhardt (270), Hedger and Hales (113), and Hutson (248)]. While the ED2^+^ subset forms the majority of the macrophages expressed in the rat testis, a significant proportion of ED1^+^ ED2^−^ cells are also present, representing about 15–20% of the population. These are assumed to be circulating “inflammatory” monocytes or recently arrived macrophage. While the ED2^+^ cells do not participate in the inflammatory response, it is clear that an influx of ED1^+^ monocytes during acute or chronic inflammation shift the cytokine balance toward and inflammatory response with the potential to overcome the immune privilege (271–273). Interestingly, it has been shown that in models of acute inflammation, the testis appears to possess a mechanism capable of counterbalancing the influx of ED1^+^ monocytes, as the increase observed is only temporary and resolved after 1–2 days (271). This mechanism is unable to counter-balance the inflammation caused by chronic infection, however, where the increase in macrophage number persists for much longer (274). In the mouse testis, a sub-population of macrophages has been identified that are capable of expressing high levels of TNFβ and appears to exhibit a tolergenic phenotype (275). In contrast to those identified in the rat testis, these macrophages have been recognized as having immunosuppressive qualities, as demonstrated by their inability to induce T-lymphocyte proliferation and their reduced antigen presenting activity (275).

### Adaptive immunity

Although the innate immune response is important as a first line of defense against *Chlamydia* infection, the adaptive response is necessary in limiting the spread of the infection, and in providing protection against recurrent infections. The adaptive, or acquired, immune response involves creating immunological memory after the initial response to the pathogen, which leads to an enhanced response in the case of subsequent exposure to the same pathogen. There are two cell types that regulate this immune response: T-lymphocytes and B-lymphocytes. Both cell types rely on their ability to distinguish between “self” and foreign antigens presented on the cell surface, a process that is regulated by the MHC. The B-lymphocytes, or B cells, regulate the humoral immune response, producing antibodies against foreign antigens. The T-lymphocytes, or T cells, regulate the cell-mediated immune response, and can be divided into sub-types. CD8^+^ T cells are also known as cytotoxic T cells and work to induce death in infected host cells. These cells predominately recognize foreign antigens presented on the cell surface by class I MHC. CD4^+^ T cells or helper T cells can be divided once again into two sub-types: Th1 cells, which induce the production of cytokines such as IFNγ. Release of IFNγ activates macrophages and induces B cells to make opsonizing and complement fixing antibodies. Th2 cell are characterized by the release of Interleukin 4 (IL-4), which in turn activate B cells to make non-cytolytic antibodies leading to humoral immunity. These cells recognize foreign antigens presented by class II MHC.

#### T cells

The involvement of T cells in the immune response against chlamydial infection was first demonstrated when Rank and colleagues observed that immunodeficient mice developed chronic *C. muridarum* infection following intravaginal inoculation, while their wild-type counterparts were able to clear the infection within 20 days (276). T cells unable to recognize pathogens or antigens without the help of APC, such as DC or macrophages. Once these cells phagocytose chlamydial EB, or infected host cells containing RBs, they degrade the chlamydial components and present the peptides via the MHC class I/II – antigen complexes. Studies in both human and mice have shown that both CD4^+^ and CD8^+^ cells are present at the site of chlamydial infection (238, 277–279). Both types of T-cell have been shown to recognize *C. trachomatis* antigens, including outer membrane protein 2 (OMP2) (280), polymorphic outer membrane protein D (POMP-D) (281), MOMP, heat shock protein 60 (hsp60) (282–284), chlamydial protease activating factor (CPAF) (285), pmpG, PmpF, and RpIF (286, 287). Upon *C. trachomatis* infection, CD4^+^ cells become activated, begin to proliferate and migrate to the genital mucosa (286, 288–292). These T-cells exhibit a characteristic Th1 response, secreting large amounts of IFNγ required to aid in clearing bacterial infection (290, 293). Previous studies have shown that infection of CD4^−∖−^ mice with *C. trachomatis* results in higher infection load during primary infection, in addition to diminished protection from secondary infection (290). However, there has been controversy regarding the protective immunity that CD4^+^ T cell memory provides following *C. trachomatis* infection (290, 294). Indeed, one such study utilized antibodies to deplete CD4^+^ T cells and showed that previous infection with *C. trachomatis* does not induce strong protective immunity upon secondary infection, and that CD4^+^ T cells are not essential for clearance of infection (294). Conversely, there have been many studies highlighting the important of CD4^+^ T cells in providing protective immunity against bacterial infection (295, 296). More recently, a study compared the ability of wild-type mice and B cell-deficient mice to clear *C. trachomatis* genital infection, and demonstrated that CD4^+^ T cell immunity was essential for protective immunity to secondary infection (297). Evidence from Gondek and colleagues support this, whereby they demonstrated that infection of the upper genital tract with *C. trachomatis* induces a robust *Chlamydia*-specific CD4^+^ T cell response that is both necessary and sufficient to clear infection and provide protection against re-infection (298). The role of CD8^+^ T cells in aiding in clearing a bacterial infection was first described in studies using a mouse model whereby splenic CD8^+^ T cells could specifically lyse *Chlamydia*-infected fibroblasts. In addition to this, CD8^+^ cytotoxic T cells induced partial protection when adoptively transferred into infected mice (288, 299, 300). CD8^+^ cells control infection of intracellular pathogens through a number of mechanisms, including cytotoxicity via granule exocytosis, production of anti-microbial peptides, and production of cytokines and chemokines. It is assumed, based on these abilities that CD8^+^ T cells function by lysing infected cells and depriving the pathogen of its intracellular environment, as well as releasing inflammatory mediators that render developing bacteria non-infectious, and recruiting or activating other immune cells to limit the survival of the pathogen (301). Although both CD4^+^ and CD8^+^ T cells contribute protective immunity during *Chlamydia* infection, differences exist depending on the model of *Chlamydia* and the mode of infection studied. For example, depletion of CD8^+^ but not CD4^+^ T cells in immune mice diminishes immune protection upon challenge with *C. psittaci* (302).

In contrast, in a model of *C. trachomatis* infected mice, depletion of CD4^+^ T cells abrogates protection more significantly compared to a decrease in CD8^+^ T cells (303, 304). It is important to note that CD4^+^ T cells are often required for the induction and preservation of a functional CD8^+^ T cell response, and in their absence both CD4^+^ and CD8^+^ T cell effector functions are severely impaired (301). T cells comprise approximately 15% of the total leukocyte population in the interstitium of rodent testis (118, 305), with CD8^+^ cytotoxic T cells predominating over CD4^+^ T cells (306). Unfortunately, very little is known about the function that T-lymphocytes carry out in the testis although there is evidence that activated memory T cells, which would normally affect an immunological response, are instead targeted for destruction when they enter the testicular environment (307, 308).

#### B cells and antibodies

The importance of B cells and the antibodies they produce in mediating immunity against *Chlamydia* infection was demonstrated more than four decades ago, when it was observed that the presence of *Chlamydia*-specific antibodies correlated with reduced rates of infection in human beings (309, 310). Later, studies went on to demonstrate that monoclonal antibodies directed against the primary *Chlamydia* antigen MOMP could neutralize infection *in vitro* (311, 312), in addition to being moderately affective at providing immunity when passively administered to mice (313). Numerous *Chlamydia* proteins have also been shown to induce antigen-specific antibodies (314). There are several mechanisms by which B cells are able to modulate immunity during *Chlamydia* infection. The first and most predominate mechanism is through antibody-mediated neutralization, whereby the B cell produces specific antibodies directed against chlamydial peptides (315). Secondly, through antibody-dependent cellular cytotoxicity that targets cells that have antibodies attached to their surface for lysis (316). Finally, B cells aid in the formation of antibody–antigen complexes that bind to receptors on APC. These then enhance phagocytosis and antigen presentation to CD4^+^ T Cells (317). Until recently, it has been thought that B cells were predominantly important in controlling secondary chlamydial infection but their presence for clearing primary infection was not essential. This was based on evidence form Su and colleagues showing that B cell-deficient mice control *Chlamydia* infection as efficiently as wild-type mice but had delayed clearance of secondary infections (318, 319).

Latterly, it was observed that B cell-deficient mice depleted of CD4^+^ T cells are unable to control secondary chlamydial infection in contrast to mice who were devoid of CD4^+^ T cells alone were able to clear the secondary infection after only a slight delay (320). This has since been attributed to the ability of B cells to produce specific antibodies, as passive transfer of immune serum or anti-*Chlamydia* antibodies into B cell-deficient, CD4^+^ depleted mice rescues their ability to clear a secondary infection (321). Moreover, the susceptibility of these B cell-deficient, CD4^+^ depleted mice to *Chlamydia* infection suggests a relationship between CD4^+^ T cells and B cells in providing protective immunity. Further to this, a study by Li and colleagues proposes that it is the antibody-producing ability of the B cells, and not antigen presentation that is responsible for the containment of bacterial infection, as CD4^+^ T cell priming was markedly reduced in B cell-deficient mice, and *Chlamydia* were unable to disseminate as far compared with controls. Interestingly, these studies demonstrate a potential role for B cells in regulating local T cell activation and bacterial dissemination during primary *Chlamydia* infection (322). Under normal conditions, B cells are very rarely detected in rat testis (255), and as such the specific role that B cells and antibodies play in controlling bacterial infections in the testis remains unknown.

### Persistence and avoiding the immune response

Persistence is defined as a long-term association between *Chlamydia* and the host, in which the organism remains viable but in a culture-negative state. The exposure of *Chlamydia* to cellular and molecular stresses is the most common cause for the bacteria to enter into a state of persistence. This state is normally characterized by the existence of large pleomorphic RBs, termed aberrant bodies (ABs), which are non-infectious, viable, and thought to be clinically undetectable. One of the defining features of the ABs, as opposed to dying or dead RBs, is that the removal of the stressor often allows the *Chlamydia* to re-enter the normal developmental life cycle. The transition of RBs into ABs can be induced *in vitro* by treatment with antibiotics, deprivation of amino acids, heat shock, or pro-inflammatory cytokines (323). Treatment of *Chlamydia* with antibiotics, such as penicillin or erythromycin, arrests chlamydial development by blocking the conversion of RBs into EBs, as shown when McCoy cells infected with *C. trachomatis* were treated with erythromycin 12 h post-infection, and developed abnormally large RBs (324).

In addition to this, it was more recently observed that addition of ampicillin to culture media of *C. pneumoniae*-infected HeLa cells resulted in the formation of aberrant, giant RBs (325). Interestingly, when compared with *C. trachomatis* infection of the same cell type, the *C. pneumonia* took much longer to recover and re-enter a normal developmental cycle (325). The reason for this remains unclear, although it is intriguing as *C. pneumoniae* are normally recognized for being very efficient at reactivation following the removal of other types of stressors, such as tryptophan-depletion, or co-culture with IFNγ (see below) (326). It has been proposed that these studies give an indication toward the possibility that inadequate microbial therapy may allow *Chlamydia* to persist *in vivo*.

In contrast to the mechanism in place for inducing persistence with antibiotics, depletion of amino acids from the culture medium of *Chlamydia*-infected cells arrests the development of both the bacteria and the host cell until such a time as the amino acids are re-introduced back into the system (327). Interestingly though, re-introduction of the amino acids does not results in full recovery of *Chlamydia* development, with smaller particle sizes observed (328). While the progressive depletion of all amino acids causes abnormal development, the depletion of tryptophan is particularly important. Most species of *Chlamydia* regulate tryptophan for survival (329). As described previously, IFNγ induces the expression of the cellular tryptophan-degrading enzyme indoleamine 2,3-dioxygenase (IDO). In the majority of *Chlamydia* species, lack of this essential amino acid results in death. However, some species have instead adapted to this mode of starvation by reverting to a persistent state. Depletion of nutrients other than amino acids has also been shown to contribute toward inducing persistence, with examples including the removal of glucose from *C. trachomatis* in McCoy cells resulting in temporary loss of infectivity and an abnormal morphology comparable to that observed in amino acid depletion (330).

The form of nutrient-depletion that has been the best characterized, however, has been the depletion of iron. Exposure of *C. trachomatis* to iron chelators causes significant morphological changes that were distinct even from those observed in other persistence systems (331). Importantly, addition of iron-saturated transferrin is able to rescue the infectivity of the *Chlamydiae*. Exposure of *Chlamydia* in culture to moderate levels of the cytokine IFNγ induces the formation of large ABs (328, 332), as described in other persistence models although with different growth characteristics (333) and altered or decreased expression of predominant chlamydial proteins or constituents such as LPS, the 60 kDa outer membrane protein (OMP) and the MOMP (334). The mechanism of action is through the activation of IDO by IFNγ, resulting in tryptophan depletion (see above) (202). As with other models, the addition of tryptophan into the culture results in reactivation of the developmental cycle. Interestingly, observations recorded by Caldwell and colleagues suggest that each species of *Chlamydia* has its own pattern of resistance to the inhibitory effects of IFNγ, and this relates directly to polymorphisms in tryptophan synthesis genes (335). For example, ocular serovars of *Chlamydia* possess a non-functional tryptophan synthase and are consequently unable to produce tryptophan of their own accord, which would make them more susceptible to inhibition by IFNγ. Genital serovars, however, have a functional tryptophan synthase, and are capable of using indole as a substrate for tryptophan synthesis (335). Persistence has also been demonstrated *in vivo*, with electron microscopic visualization of morphologically aberrant *Chlamydia* forms in diseased tissue. Nanagara and colleagues showed that atypical, pleomorphic AB with poorly defined outer membranes dominated within infected fibroblasts and macrophages from patients with *C. trachomatis*-associated reactive arthritis (336). In addition, miniature *C. trachomatis* forms have been observed in total ejaculate and expressed prostate secretions from patients with chronic chlamydial prostatitis (337) and in the oviducts of mice experimentally infected with *C. trachomatis* (338). Importantly, there is also evidence for the ability of *Chlamydia* to become reactivated *in vivo*. A study performed by Dean and colleagues demonstrated the individuals experiencing recurrent infections possessed chlamydial isolates of the same genotype (339). Unfortunately, this study could not make allowance for recurrence from infected partners. However, an additional study did show a 10% recurrence of genital *C. trachomatis* infection in individuals that reported abstinence or complete condom use following treatment with antibiotics (340).

It is well understood that *Chlamydia* utilizes the state of persistence not only to continue its infectious life cycle but also as a means of evading the host immune response. However, *Chlamydiae* have also developed additional means of remaining undetected. One such survival strategy employed by both *C. trachomatis* and *C. pneumonaie* is to inhibit apoptosis of the host cells (341, 342), which ensures that host cell lysis does not occur prior to the completion of the bacterial developmental cycle. It is also thought that inhibition of apoptosis may limit the number of apoptotic infected cells available to APC, and allow infected cells to resist being killed by effector CD8^+^ T cells (301). Nevertheless, the influence that *Chlamydia* has on host cell apoptosis is complex and controversial, as studies have also shown *Chlamydia* is also able to induce apoptosis (343).

The induction of apoptosis is thought to occur in two ways: firstly through the expression of *Chlamydia* proteins associated with death domains, which may promote apoptosis through association with mammalian death receptors (344, 345). Although it appears counter-intuitive for *Chlamydia* to both induce and suppress apoptosis, it may help protect the bacterium from the immune response *in vivo*. As mentioned previously, it has been proposed that *Chlamydia* may restrict apoptosis in order to complete the developmental cycle, allowing RBs to convert back into infectious EBs. Once the developmental cycle nears completion, it may be beneficial for the bacterium to induce apoptosis in order to avoid necrosis, which is known to stimulate inflammation and subsequently enhance *Chlamydia*-specific immune response. It may also aid in the release of infectious EBs into the cytosol (346). In addition to this, it has been proposed that *Chlamydia* may down-regulate MHC class I and II expression on infected cells in order to evade T cell-mediated immune recognition. This is evidenced by the secretion of a chlamydial protease-like activity factor (CPAF) into the cytosol of *C. trachomatis* or *C. pneumonaie* infected cells, which then works to degrade specific host cell transcription factors that control the constitutive and IFNγ-inducible expression of MHC class I and II molecules, respectively (347–349). Down-regulation of MHC expression on infected cells has the potential to limit their recognition by CD4^+^ and CD8^+^ T cells, in addition to rendering the infected cells susceptible to lysis by NK cells. Evidence for *Chlamydia*’*s* ability to evade the immune system *in vivo* is indirect. It is clear that previous exposure of human beings to *C. trachomatis* does not provide robust immunity against re-infection, and this may be due to poor development of an adequate immune response (8). Studies performed in mice have demonstrated that *Chlamydia*-specific T cells do not appear to develop into memory T cells capable of mounting a robust recall response when compared with infection with well-known pathogen such as *vaccinia* (8).

### Chronic inflammation

Following chronic or recurrent genital tract infection with *C. trachomatis*, the chronic inflammation that develops can lead to tubal scarring, ectopic pregnancy, and infertility in women (350), as well as epididymitis, prostatitis, and orchitis in men (see above). As discussed above inflammation is primarily caused by the activation of macrophages during the immune response, resulting in the release of different mediators that perpetuate the pro-inflammatory response such as IL-1, TNFα, and prostaglandins. While this pathway is thought to result in the majority of the tissue damage associated with the pathology of *Chlamydia* infection, a number of other factors may also contribute to the etiology.

The first of which is the presence of ROS. ROS comprises several oxidizing, oxygen-containing species that are generated in a physiological system through one-electron-transfer reactions during metabolism, and include hydroxyl radical (OH^−^), superoxide anion radical $\left(O_{2}^{-}\right)$ and hydrogen peroxide (92). ROS are known mediators of the immune response, and work by promoting endothelial dysfunction through the oxidation of crucial signaling proteins (351). Importantly, testicular macrophages are known to produce ROS during infection and in response to LPS (352), and LPS is known to induce oxidative stress (353–355). The release of ROS by activate macrophages not only affects invading pathogens, but may also expose adjacent host tissue and cells to oxidative stress. It has been proposed that Leydig cells may be particularly susceptible to extracellular sources of ROS during immune responses due to their close proximity to interstitial testicular macrophages (356, 357). In addition to this, as ROS produced by *C. trachomatis* infection could be an important factor in the damage of sperm cells, it is thought that low molecular weight and enzymatic secretions of the male genital tract may play an active role in suppressing the deleterious effects of ROS, leading to the preservation of fertility (102, 358). ROS such as superoxide anion are able to rapidly combine with NO to form reactive nitrogen species (RNS). The RNS in turn induces nitrosative stress, which adds to the pro-inflammatory burden of ROS (359). In addition to this uncontrolled NO production alone has been implicated in chronic inflammation during *Chlamydia* infection (360, 361).

In addition to the presence of ROS, the presence of small anti-bacterial molecules in the male reproductive tract may also contribute to a sustained inflammatory response. Defensins are small, positively charged peptides that disrupt bacterial infection by forming multimeric pores in the pathogens membrane (186). A large majority of epithelial cells produce beta defensins, including those of the epididymis and testis, with a large number of epididymal-specific beta defensins being identified in both the mouse and rat (362–367). Most of these epididymal defensins are developmentally or hormonally regulated, with evidence that some might play a role in sperm maturation (362, 365). Normally, epithelial defensing production is stimulated by TLR activations and cytokines during inflammation, with studies showing that LPS induces mRNA expression of defensin and defensin-like spag11, as well as pro-inflammatory cytokines in the rat epididymal caput, cauda and testis (368). Importantly, it has been shown recently that stimulation of mouse macrophages with beta defensin 14 results in the synergistic and enhanced expression of pro-inflammatory cytokine and chemokines induced by TLR ligand re-stimulation (369). It is, therefore, possible that infection with *Chlamydia* may stimulate the up-regulation of beta defensins in the male reproductive tract, which may in turn activate mouse macrophages to produce an inflammatory response. Therefore, chronic, or recurrent chlamydial infection may lead to chronic inflammation, and subsequent tissue and cell damage.

## Conclusion

*Chlamydia* infection is a public health concern, with the development of a vaccine capable of preventing infection without activating harmful immune responses the best solution for controlling this sexually transmitted disease. Unfortunately, as yet, no vaccine has been developed that has been able to block infection, highlighting the dynamic and complex nature of the immunobiological response that is mounted against the invading bacteria. Clearance of chlamydial infection requires the coordinated action of both the innate and adaptive immune systems, with multiple levels of cross-talk and redundancies in place. The problem is compounded even more in regions of the male reproductive tract, particularly the testis, whose blood–testis barrier allows the cells within to mature and function without risk of attack from the immune system. However this barrier, also prevents a normal immune response from occurring, and the underlying mechanisms of this response remain unclear. It is also unknown how the presence of the blood–testis barrier would affect the delivery of an effective vaccine. Therefore, research aiming to provide a greater understanding of the role and regulation of the immune response within the male reproductive tract would aid in the development of *Chlamydial* vaccine.

## Conflict of Interest Statement

The authors declare that the research was conducted in the absence of any commercial or financial relationships that could be construed as a potential conflict of interest.
